# Human papilloma virus is not detectable in samples of urothelial bladder cancer in a central European population: a prospective translational study

**DOI:** 10.1186/s13027-015-0028-7

**Published:** 2015-09-21

**Authors:** Sebastian C. Schmid, Leonore Thümer, Tibor Schuster, Thomas Horn, Florian Kurtz, Julia Slotta-Huspenina, Judith Seebach, Michael Straub, Tobias Maurer, Michael Autenrieth, Hubert Kübler, Margitta Retz, Ulrike Protzer, Jürgen E. Gschwend, Dieter Hoffmann

**Affiliations:** Department of Urology, Klinikum rechts der Isar der Technischen Universität München, Munich, Germany; Department of Virology, Technische Universität/Helmholtz Zentrum München, Munich, Germany; Institute of Medical Statistics and Epidemiology, Technische Universität München, Munich, Germany; Institute of Pathology, Klinikum rechts der Isar der Technischen Universität München, Munich, Germany; http://www.mriu.de

**Keywords:** Bladder cancer, Urothelial carcinoma, Papillomavirus, HPV, PCR, Polymerase chain reaction

## Abstract

**Background:**

Previous investigations on the association of human papillomavirus (HPV) and human bladder cancer have led to conflicting results. The aim of this study was to determine if low and high risk HPV play a role in the etiology of superficial low grade and invasive high grade urothelial carcinoma of the bladder.

**Methods:**

We prospectively collected tumor samples of urothelial carcinoma of the bladder from 109 patients treated with transurethral resection or cystectomy, with bladder tissue from transurethral resection of the prostate serving as control. Unfixed, frozen tumor samples were analyzed for the presence of 14 high risk HPV types using real time PCR. Additionally, all specimens were tested for 35 low risk HPV types with a conventional PCR using degenerate primers located in the L1 region. Six frozen samples of cervical carcinoma served as positive controls.

**Results:**

We included 109 cases of bladder cancer with 41 superficial (pTa low grade) tumors, 56 invasive (pT1-T4) high grade tumors and 12 others (pTa high grade + pTis). We have not detected HPV-DNA in any sample (95 % Confidence Interval [CI] 0–3.3 %), superficial tumors (95 % CI 0–6.4 %) or in invasive tumors (95 % CI 0–8.6 %) with correct positive controls.

**Conclusions:**

Using a broad, sensitive assay with prospectively collected specimens of a Central European population we could not detect HPV-DNA in any of the cases. Our results suggest that it is unlikely that HPV infections play a major role in the development of urothelial bladder cancer.

## Introduction

Bladder cancer is the fourth most common malignancy of males and the eighth most common of females and leads to estimated 170,000 deaths per year worldwide [1]. Bladder cancer shows urothelial differentiation in more than 90 % of cases in Western countries. It can be separated into two major entities. On the one hand there are superficial, low grade (G1) tumors, which are relatively benign but do often recur, on the other hand there are invasive, high grade (G3) tumors, which have a high risk of progression, metastasis and cancer-related death.

Several risk factors have been identified like tobacco use as well as occupational or environmental exposure to noxious substances like aromatic amines [2]. So far, prevention strategies are limited to the elimination of risk factors while treatment of bladder cancer is invasive and intensive in terms of costs and time [3]. Additionally, treatment is further hindered by the lack of good markers for prognosis and therapy response [4]. The WHO estimated that about 12 % of worldwide cancer cases are caused by viral infections [5]. Thus, identification of an infectious agent like human papilloma virus (HPV) in the etiology of bladder cancer might have a relevant impact on prevention and therapy. HPV is a DNA virus which belongs to the papillomavirus family. It has been shown that HPV plays a crucial role in the development of cervical carcinoma [6] and is also linked to anal, vaginal, penile and oropharyngeal tumors. HPV is a highly-divergent virus with more than 150 types, which are grouped into high risk and low risk according to their potential to induce malignant transformation [7].

It is known for cattle that the infection with bovine papillomavirus (BPV) in combination with certain food components leads to bladder cancer [8]. On the contrary, the role of HPV in human bladder cancer is unclear. Published studies show inconsistent results concerning HPV association in bladder cancer, with a prevalence from 0 % [9–12] to 35–52 % [13–15]. This might be due to low case numbers, diversity of patients and analyzed tumor entities and restriction of the analysis to a subset of HPV types. Another issue is potential contamination, leading to false positive results, which can for example occur during the cutting process of formalin fixed, paraffin-embedded tissue, sample preparation or DNA analysis.

**Fig. 1:**
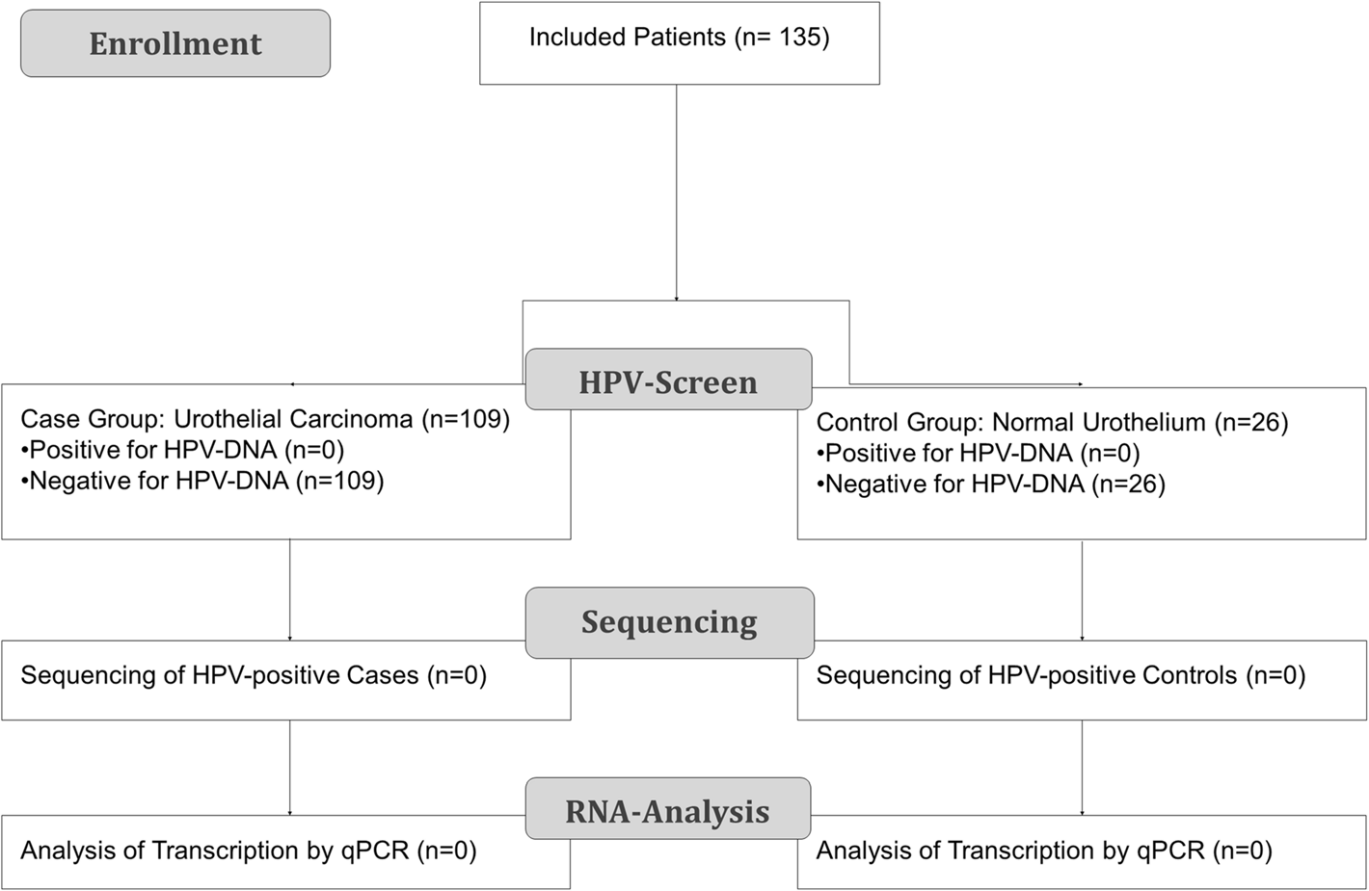
Flow chart describing study design and enrollment. Because of the negative result in the screening phase, planned sequencing and transcription analysis of positive cases could not be performed

We aimed at clarifying the role of HPV in the etiology of urothelial cancer and therefore conducted a systematic study analyzing sufficient case numbers and carefully controlling logistic and technical biases (see Fig. 1). To this end, we analyzed the presence of HPV-DNA in superficial low grade and invasive high grade urothelial cancer. We used highly sensitive PCRs to detect a broad range of HPV types in prospectively acquired, fresh frozen tumor samples.

## Methods

### Tissue sample collection

The study was performed in a prospective manner. A complete study protocol was written before start of the study. The study was approved by the ethics commission of the faculty of medicine of Technische Universität München before start of enrollment. All patients signed a written informed consent prior to study entry.

Patients undergoing cystectomy or transurethral resection of the bladder (TURB) at the Department of Urology, University Hospital rechts der Isar of the Technische Universität München, were enrolled. Only patients with histologically confirmed urothelial carcinoma of the bladder were included and classified according to TNM-system (UICC 2010). Patients with benign prostate hyperplasia served as controls, who underwent transurethral resection of the prostate (TURP) at the same hospital and had no history of urologic neoplasia. We chose this patient group for two reasons: First, TURP is performed for a non-neoplastic condition. Second, it is possible to get bladder mucosa samples during the necessary operation, whereas surgery in healthy controls for study reasons only is ethically not justifiable.

Tissue samples of bladder cancer were collected during cystectomy or TURB. Samples were dissected under sterile conditions and immediately transferred to RNAlater solution (Qiagen, Germany) and stored at −80 °C until further processed for the extraction of nucleic acids. Of each sample, a formalin-fixed part was used for histological analysis to confirm malignancy. Samples of normal bladder mucosa were obtained during TURP and handled in the same way as the tumor samples, as well as six samples of cervical carcinoma which served as positive controls for the validation of extraction and detection methods.

### DNA Extraction

Tissue samples of 3×3 mm in size were homogenized in 700 μl lysis buffer using the Qiagen TissueLyser. DNA was extracted with the Abbott m2000sp instrument in 500 μl sample volume and 70 μl eluate volume according to the manufacture’s protocol. Positive and negative control samples were extracted in the same run. For positive control samples we used HPV positive tissue from fresh frozen cervical carcinoma to control overall sample handling, DNA extraction from tissue samples and HPV detection. Cloned mouse prion protein gene was added to each sample to control extraction efficiency.

### PCR

HPV-DNA was detected by PCR amplification. For high risk HPV genotypes (16, 18, 31, 33, 35, 39, 45, 51, 52, 56, 58, 59, 66, 68) we used the Abbott Realtime HR HPV assay using fluorescent probes and running on the m2000sp instrument (Abbott, Wiesbaden, Germany). Three forward and two reverse primers target the conserved L1 region. According to the manufacturer, the analytical sensitivity depends on the subtype: 500 copies per assay for 16, 18, 35, 39, 45, 51, 59, 66, 68; 2000 copies per assay for 31, 33, 52, 56; and 5000 copies per assay for 58. HPV-DNA positive solutions as well as SiHa cells (kindly provided by Dr. S. Silling of the “Nationales Referenzzentrum für Papillom- und Polyomaviren”, Köln, Germany) harbouring 1–2 HPV copies per cell were used as controls for analytical sensitivity. The endogenous human beta globin gene (amplicon size 136 nucleotides) served as a control for cell adequacy, sample extraction and amplification efficiency.

Additionally we designed a nested conventional PCR for broad detection of low risk HPV. To this end, nucleotide sequences of 35 low risk HPV genotypes (6, 7, 11, 13, 30, 32, 34, 40, 42, 43, 44, 53, 54, 55, 57, 61, 62, 67, 69, 70, 72, 73, 74 81, 82, 83, 84, 85, 86, 87, 90, 91, 97, 102, 106) were obtained from NCBI and were aligned with DNAMAN (Lynnon Corporation, Canada). Sequences in the L1 region showing high homologies were selected for primer design, leading to an amplicon of about 387 (380–390) nucleotides in length. Ten forward primers and three reverse primers were designed to be highly specific for selected HPV types. Inosine was used as a degenerate base at up to two positions, tolerating up to one mismatch (Table 1). We chose primers with similar melting temperatures and with low primer hybridization, self-annealing and mispriming according to DNAMAN.

**Table 1:** Primers used in the multiplex PCR for 35 low-risk HPV types using IUPAC 1-letter code with I for desoxyinosines as degenerate bases

Primer	Sequence
Forward A	5'-AGG ATG GIG ACA TGG TGG A-3'
Forward B	5'-AGG ATG GTG ACA TGG TAG A-3'
Forward C	5'-AGG ATG GCG ACA TGG TTG A-3'
Forward D	5'-AGG ATG GGG ACA TGA TAG A-3'
Forward E	5'-AGG ATG GGG ACA TGA TTG A-3'
Forward F	5'-AGG ATG GIG ATA TGG TGG A-3'
Forward G	5'-AGG ATG GIG ATA TGG TAG A-3'
Forward H	5'- AGG ATG GIG ATA TGG TTG A-3'
Forward I	5'-AGG ATG GIG ATA TGA TAG A-3'
Forward J	5'-AGG ATG GIG ATA TGA TTG A-3'
Reverse A	5'-CAG GGI CAI AAC AAT GG-3'
Reverse B	5'-CAG GGI CAI AAT AAT GG-3'
Reverse C	5'- CAA GGI CAC AAT AAT GG-3'

For validation of our in-house PCR, HPV-DNA isolated from patient swabs positive for types 42, 53, 55, 44, 57, 61, 40 and 30, 33 were PCR amplified, cloned and sequenced. As standards we used plasmids with inserted type 42 and 57 sequences. After TOPO cloning and propagation in *E. coli* plasmid preparations were quantified and copy numbers were determined based on optical density and molecular weight. Analytical sensitivity was determined to be below 100 copies per reaction. The PCR mix contained 5 μl of DNA samples, 1× reaction buffer, 0.3 Taq polymerase (Qiagen), 20 pmol of each primer (2 μl of 10 μM), 10 nmol of dNTP (2 μl of 5 mM) and water to a final volume of 50 μl. A median amount of 109 ng DNA was added into each PCR reaction. PCR conditions were the following: initially 1 activation cycle at 94 °C for 15 min, 45 cycles of denaturation at 95 °C for 30 s, annealing at 46 °C for 30 s, elongation at 72 °C for 30 s, and a final elongation cycle at 72 °C for 7 min. Amplification products were visualized under UV-light after electrophoresis in a 1.5 % agarose gel, and amplicons of the correct size were cloned and genotyped by sequencing.

Statistical planning and analysis was performed using R Software (www.r-project.org) using continuity-corrected confidence intervals for the difference between two independent proportions [16].

## Results

Our study included 109 patients with a median age of 73 years (range 45–94), 81 % were male. Five patients had been excluded due to non-urothelial cancer in the final pathology assessment. Of the 109 cases with urothelial cancer, 41 had superficial (pTa low grade) urothelial cancer, 56 invasive (pT1-T4) high grade urothelial cancer and 12 other urothelial tumor types (pTa high grade or pTis) (see Table 2).

**Table 2:** Patient characteristics

Patient characteristics			
Age (years)	Median	Range	
Case group	73	45-94	
Control group	68	53-86	
	Male	Female	Overall
Case group	88 (81 %)	21 (19 %)	109
Control group	26 (100 %)		26
Histopathologic results (Case group)	Male	Female	Overall
Low grade (G1)			
pTa low grade	34	7	41 (38 %)
High grade (G3)			
pTa/pTis	10	2	12 (11 %)
pT1	9	3	12 (11 %)
pT2	11	4	15 (14 %)
pT3	17	4	21 (19 %)
pT4	7	1	8 (7 %)
Non urothelial differentiation (excluded)	5	0	5

Fresh tissue samples obtained during tumor resection or TURP in control patients were subjected to DNA extraction and PCR analysis for HPV. For high risk HPV screening, we used the commercial Abbott test detecting the following genotypes: 16, 18, 31, 33, 35, 39, 45, 51, 52, 56, 58, 59, 66, 68. In order to detect a wide range of low risk HPV genotypes, we developed a broad multiplex PCR detecting at least the following 35 mucosa-associated HPV genotypes: 6, 7, 11, 13, 30, 32, 34, 40, 42, 43, 44, 53, 54, 55, 57, 61, 62, 67, 69, 70, 72, 73, 74 81, 82, 83, 84, 85, 86, 87, 90, 91, 97, 102, 106 at high sensitivity.

We detected no HPV-DNA in any urothelial cancer sample (95 % confidence Interval [CI] 0–3.3 %), superficial tumors (95 % CI 0–6.4 %) or invasive tumors (95 % CI 0–8.6 %) although for each run positive controls were detected (sensitivity ≤100 copies). All urothelial tissues from the control group of patients with benign prostate hyperplasia (*n* = 26) were negative for HPV-DNA. The respective 95 % continuity corrected confidence intervals for the difference in proportions (cases – controls) were −16.0 to +4.3 %, −16 to +8.0 % and −16.0 to +10.7 %, the negative values corresponding to the expectable difference in the prevalence of HPV in the control group (TURP). Five of six cervical carcinoma tissues were positive for high risk HPV.

SiHa cells harbor only 1–2 copies of HPV16 per cell. Thus we used them to confirm the sensitivity of our test procedure. DNA of approximately 5×10^6^ cells was extracted with DNA Mini Kit (Qiagen, Germany) and eluted in 200 μl, equaling 25,000 cells per μl. An in house PCR detecting human prion protein to estimate the genomic DNA content of a sample yielded an equivalent of 32,000 cells per μl. We then tested a serial dilution, starting with 1:10 and an input of 5 μl (corresponding to 10^5^ cells). We detected HPV 16 DNA down to 10 cells per assay (1:10.000), corresponding to 10–20 HPV copies per assay. This is approximately 1/1000 of the cell number which we analyzed in our clinical samples. In conclusion our method is able to detect HPV with a high sensitivity also in cells with low copy numbers (1–2 copies per cell). With a DNA amount equaling a cell count of >10,000 cells per assay, both our in-house as well as the commercial Abbott assay were able to detect ≤1 HPV copies per cell.

## Discussion

In this study we analyzed two pathological subgroups of bladder cancer for the presence of various high and low risk HPV subtypes. Superficial low grade and invasive high grade bladder cancer show very distinct characteristics in regard to molecular alterations (like *FGFR3* or *TP53* mutations) and clinical behavior and might represent different etiologic entities [17]. In our population we detected no HPV-DNA in bladder tissue, irrespective of subgroup, tumor stage or grade. Previous studies mostly focused on high risk HPV as these have been shown to cause malignant transformation due to the oncogenic nature of their E6 and E7 proteins. However, occasionally low risk HPV types were found in cancerous lesions [18], and the classification in high and low risk based on cervical cancer may not fit to all tissues. Thus we also tested for a series of low risk HPV types that are associated with mucosal infection. However, all samples tested negative for all HPV types. Five of six cervical cancer samples tested positive for HPV-DNA, confirming that our extraction method worked properly. By collecting all tissue samples prospectively and processing only frozen, unfixed samples, we on the one hand ensure high PCR sensitivity and on the other hand avoid methods prone to contamination like processing of formalin-fixed, paraffin-embedded material which might be contaminated during cutting and storage of samples. A potential limitation of our study remains the case number, which makes it unlikely, but does not allow to completely exclude HPV infections as a contributing factor to bladder cancer. As expected, urothelial differentiation was most common, limiting our conclusions to this subtype. Also, no patients below the age of 45 years were included in our group. With our methods we cannot rule out that HPV plays a role at very early cancer stages, for example with a “hit and run mechanism” as discussed by Iwasaka et al. [19]. For instance HPV could inactivate the p53 tumor suppressor gene years before tumors occur and may be undetectable at the later stages. However, HPV DNA is usually detectable in tumors where it is thought to play a role in oncogenesis [20]. Also the fact that we detected no HPV DNA in early and advanced tumor stages argue against a role of HPV in early carcinogenesis and subsequently disappearing virus. Along these lines Pichler et al. found a very low HPV prevalence in 186 retrospectively tested non-muscle invasive bladder cancer tissues [21].

Two recent meta-analyses tried to elucidate the role of HPV infection in bladder cancer [22, 23]. The authors analyzed 22 case–control studies. The majority of these included a small case number (*n* < 50) and a mixed methodology. They reported an overall prevalence of 16.9 % (95 % CI: 14.4–17.4) and a pooled odds ratio of 2.13 % (95 % CI: 1.54–2.95) resp. 2.84 % (95 % CI: 1.39–5.8) for the presence of HPV in bladder cancer tissue. The geographical distribution showed the highest prevalence in Asia (24.6 %), followed by Africa (19.4 %) followed by North America (13.5 %) and Europe (13.1 %) [23]. Both meta-analyses concluded that there is a significant but moderate association between HPV prevalence and bladder cancer. Our results do not support this conclusion, in line with results from Knowles et al. [11] and Yavuzer et al. [24]. Knowles et al. analyzed 100 urothelial carcinomas by southern blot and a general primer PCR for HPV-DNA in the 1990s in Great Britain with negative result. Yavuzer et al. tested 70 cases of urothelial carcinoma with a nested PCR in 2011 in Turkey. No HPV-DNA was found in the urothelial carcinomas, while 15/18 cervical carcinomas, serving as positive controls were positive. As the meta-analysis did neither take viral load nor PCR specificity into account, positive association could have been either caused by low PCR signals with unknown relevance or by false-positive signals.

Detection of genomic DNA, particularly in low copy numbers, shows no causal cancer relationship [25]. To confirm positivity for HPV it would have been important to analyze transcription and translation of HPV genes in PCR positive samples. Functional analysis, e.g. studying the interaction of HPV proteins with host pathways, can proof oncogenicity in relevant cell types. However, detection of HPV-DNA - as we did in our study - is still regarded the most sensitive method of detection in tumor samples.

We conclude that the absence of HPV DNA in normal bladder and tumor tissue makes a strong association of HPV with bladder cancer unlikely.
